# HDR Brachytherapy and SBRT as Bridging Therapy to Liver Transplantation in HCC Patients: A Single-Center Experience

**DOI:** 10.3389/fonc.2021.717792

**Published:** 2021-08-26

**Authors:** Franziska Walter, Frederik Fuchs, Sabine Gerum, Maya C. Rottler, Robert Erdelkamp, Jens Neumann, Lukas Nierer, Markus Guba, Enrico N. De Toni, Max Seidensticker, Jens Ricke, Claus Belka, Stefanie Corradini

**Affiliations:** ^1^Department of Radiation Oncology, University Hospital, LMU Munich, Munich, Germany; ^2^Department of Radiation Oncology, Landeskrankenhaus Salzburg, Uniklinikum der Paracelsus Medizinischen Universität, Salzburg, Austria; ^3^Department of Pathology, University Hospital, LMU Munich, Munich, Germany; ^4^Department of Surgery, University Hospital, LMU Munich, Munich, Germany; ^5^Department of Gastroenterology, University Hospital, LMU Munich, Munich, Germany; ^6^Department of Radiology, University Hospital, LMU Munich, Munich, Germany

**Keywords:** interstitial brachytherapy, stereotactic body radiotherapy, liver transplantation, bridging therapy, local ablative therapy

## Abstract

**Background:**

In the treatment of patients with HCC awaiting liver transplantation (LT), local ablative treatments (LAT) are available either for downstaging or as bridging treatment. We present our clinical experience with both available radiation-based techniques, brachytherapy (BT), and stereotactic body radiotherapy (SBRT).

**Methods:**

All patients diagnosed with HCC and who were treated with BT or SBRT at our institution between 2011 and 2018 were retrospectively reviewed. The current analysis included all patients who subsequently underwent LT.

**Results:**

A total of 14 patients (male=9; female=5) were evaluated. Seven underwent BT for bridging before LT, and seven were treated with SBRT. BT was performed with a prescribed dose of 1 × 15 Gy, while SBRT was applied with 37 Gy (65%-iso) in three fractions in six patients, and one patient was treated with 54 Gy (100%-iso) in nine fractions. The treatment was generally well tolerated. One case of grade 3 bleeding was reported after BT, and one case of liver failure occurred following SBRT. All patients underwent LT after a median time interval of 152 days (range 47–311) after BT and 202 days (range 44–775) following SBRT. In eight cases, no viable tumor was found in the explanted liver, while four liver specimens showed vital tumor. The median follow-up after SBRT was 41 months and 17 months following BT. Overall, no hepatic HCC recurrence occurred following LT.

**Conclusion:**

Both SBRT and BT are feasible and well tolerated as bridging to LT when applied with caution in patients with impaired liver function. Radiation-based treatments can close the gap for patients not suitable for other locally ablative treatment options.

## Introduction

Hepatocellular carcinoma (HCC) is the most common primary liver malignancy and mostly develops on the background of preexisting liver cirrhosis or chronic liver disease (1). The treatment of HCC is stage dependent and determined by several variables, including liver function and performance status of patients, in addition to the number and size of lesions. For patients with liver cirrhosis and unresectable early-stage HCC (BCLC A), liver transplantation (LT) is an option that provides a curative treatment of the underlying liver disease in addition to a potentially curative treatment for HCC. The prognosis of patients undergoing LT due to HCC has greatly improved after the introduction of the Milan criteria (2), which determines eligibility for LT based on the maximal number and size of HCC lesions. Specifically, a patient is suitable for transplantation if a single tumor is present and does not exceed 5 cm in size, or if up to three nodules with a diameter ≤3 cm in diameter are present (3). While patients within the Milan criteria are considered eligible for primary LT, patients outside the Milan criteria may be considered for LT based on an individual evaluation that includes the response to local ablative treatment (LAT) (4).

Limiting to the application of LT is the shortage of liver grafts resulting in a considerable delay of treatment for many patients while waiting for donor organs. Patients eligible for LT are managed on waiting lists. Priority is given, e.g., according to the model for end-stage liver disease (MELD) score. In HCC patients, bridging therapies are frequently applied to avoid tumor progression, which could result in delisting. Moreover, it was shown that successful local ablative therapy before liver transplantation is an independent statistically significant factor for long-term tumor-related survival for patients with HCC in cirrhosis (5). The American Association for Study of Liver Disease (AASLD) recommends bridging therapy in patients facing more than 6 months’ waiting time (6).

Several LATs are available as treatment options for either downstaging or bridging before LT. Most commonly, TACE or RFA are used as bridging therapies with good results (7). For a subset of patients, however, these treatment options are not ideal for reasons such as tumor size, localization, or proximity to adjacent structures. In these cases, radiation-based treatment options using either stereotactic body radiotherapy (SBRT) or brachytherapy (BT) can be more suitable approaches. Both treatments show good local control rates (8, 9). Critical for the decision to apply LAT in patients with preexisting liver cirrhosis is the liver function, which is traditionally assessed through the Child-Pugh score (CPS). So far, radiation-based local treatments are scarcely used in patient with liver cirrhosis due to concerns about radiation-induced liver disease (RILD). However, with ongoing improvements in radiation techniques such as SBRT and interstitial BT, this practice needs reconsideration.

Currently available data on the effectiveness of bridging therapies mainly derive from retrospective studies, case series, and reports on single-center experiences (10). External beam radiation therapy has only lately been introduced as a treatment option for HCC (11). Although the reported experience in the treatment of HCC is promising, only very few data are available on the use of SBRT as a bridging option for the treatment of HCC in patients on the waiting list for liver transplantation (12), and no direct comparison of the two radiation-based treatment options has been published so far. We therefore report our clinical experience with both techniques, BT and SBRT in the setting of bridging therapy prior to LT.

## Methods

We retrospectively reviewed all patients diagnosed with HCC and who were treated with BT or SBRT between 2011 and 2018 in our institution. Only patients who were listed for LT and who subsequently underwent liver transplantation were included in the current analysis. We report on patient and treatment characteristics and pathological and clinical outcome. The study was performed in accordance to the Declaration of Helsinki in its latest version and was approved by the Ethics Committee, LMU Munich (#EK-LMU-18-511, #EK-LMU-607-16).

### HDR-BT

**Figure 1:**
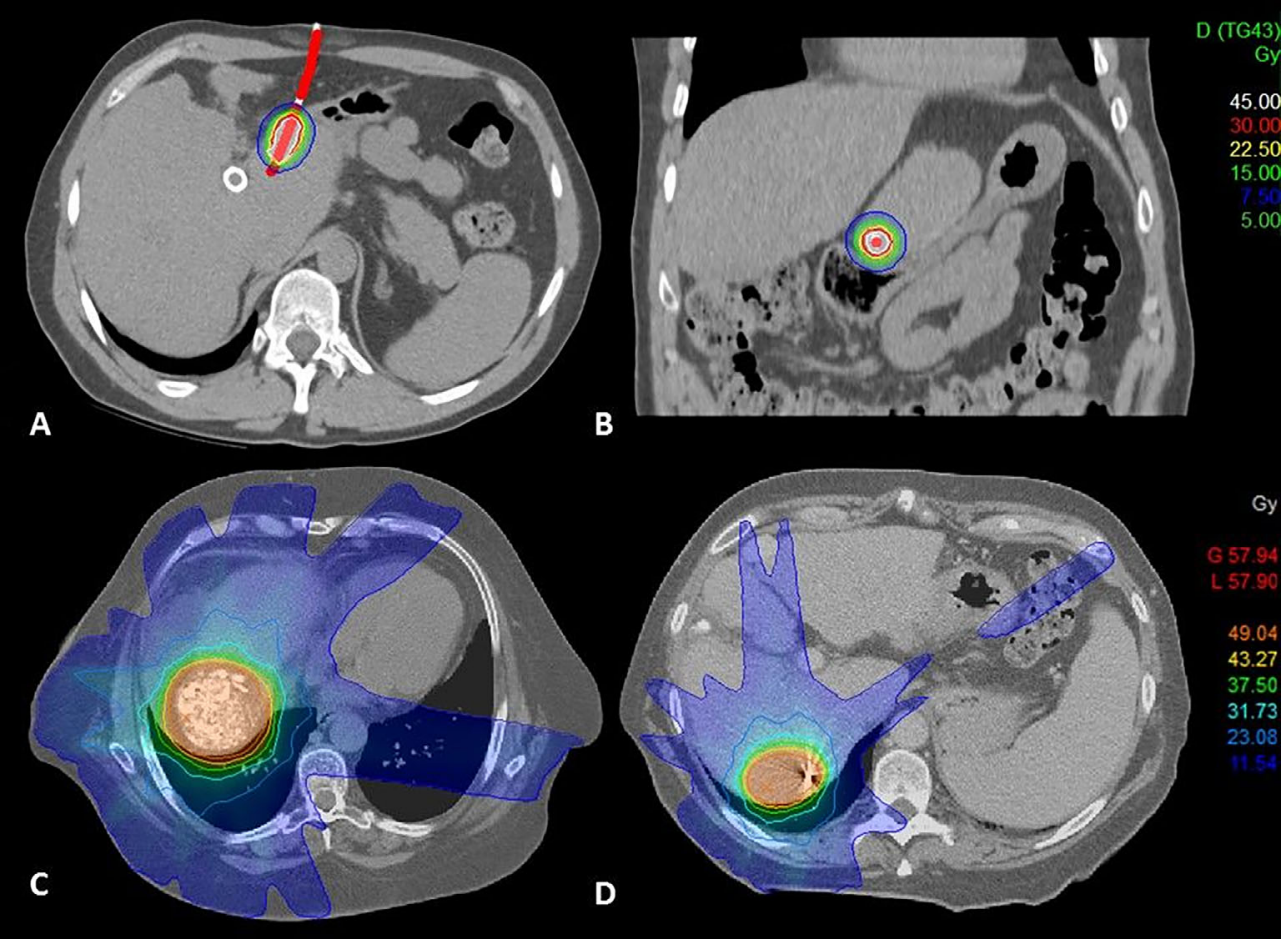
**(A)** Brachytherapy dose distribution coronal view, **(B)** Brachytherapy dose distribution sagittal view, lesion in close proximity to the stomach, **(C)** SBRT dose distribution, treatment following TACE, **(D)** SBRT dose distribution lesion with fiducial marker.

The placement of brachytherapy catheters (Primed Halberstadt Medizintechnik, Halberstadt, Germany) was performed CT-guided by an experienced interventional radiologist. A dedicated planning CT was acquired with a slice thickness of 2mm, and i.v. contrast was administered, if applicable. For treatment planning, the CT dataset was transferred to the treatment planning software Oncentra Brachy (Elekta AB, Stockholm, Sweden) version 4.5.2. The clinical target volume (CTV) and adjacent organs at risk were delineated by a radiation oncologist. The 3D catheter reconstruction was performed using the hyperdense tip marker of the BT catheter as reference point. For dose optimization, a dwell point step width of 2 mm was defined. Dose optimization was performed manually by a medical physicist using a prescribed dose of 15 Gy to 100% of the target volume (D100), example shown in **Figure 1**. Organs-at-risk dose constraints were given as follows: bowel/colon and stomach D1 ccm: 12 Gy, D0.1 ccm: 15 Gy; esophagus D1 ccm: 12 Gy, D0.01 ccm: 15 Gy; spinal cord D0.01 ccm: 10 Gy; skin D0.01 ccm: 10 Gy. One-third of the uninvolved liver was irradiated with less than 5 Gy.

### SBRT

Prior to SBRT, patients either underwent CT-guided implantation of one to three fiducial markers (Visicoil™, IBA dosimetry or MPB™, MPB Scherer Medizinprodukte) or TACE with injection of lipiodol, which provided enhancement suitable for image guidance for treatment application, example shown in **Figure 1**. A dedicated planning CT with i.v. contrast was acquired using a 4D-CT for calculation of all respiratory phases. Patient immobilization included a vacuum pillow with wing step and abdominal compression since 2014 and when tolerated. A liver-specific MRI was used for image fusion. Gross tumor volume (GTV) was delineated on free-breathing CT and in all respiratory phases. An internal target volume (ITV) was generated and isotropically expanded by 6 mm to create a planning target volume (PTV). The radiation dose was prescribed to a PTV surrounding isodose. Two dosing schemes were used, either 9 × 6 Gy (100%-isodose) or 3 × 12.5 Gy (65%-isodose). For three fractions, a mean dose of 13 Gy was allowed for the entire liver, D700 ccm < 15 Gy; stomach Dmax: 22 Gy, D10 ccm: 16.5 Gy; duodenum Dmax: 22 Gy, D5 ccm: 16.5 Gy; spinal cord Dmax: 18 Gy; kidney D200 ccm: 14.5 Gy.

### Toxicity

We assessed acute toxicity following SBRT or BT by review of treatment reports and laboratory results, specifically INR, albumin, and bilirubin. CPS at the time of treatment was compared to CPS post-treatment in all patients, if blood levels were available. Information on ascites and encephalopathy was assessed by review of imaging and medical reports.

### Patient Follow-Up

Follow-up was reviewed retrospectively. All available patient reports, surgical reports, and imaging reports were included. Local control before LT was defined as lack of local progression in the time between treatment and LT. It was determined by radiological reports on magnetic resonance imaging (MRI) or computed tomography (CT) before LT or the histopathological report of the explanted liver. Hepatic or distant failure after LT were determined according to radiological reports. Toxicity was graded according to the Common Terminology Criteria for Adverse Events (CTCAE) version 5.0.

### Histological Classification

**Figure 2:**
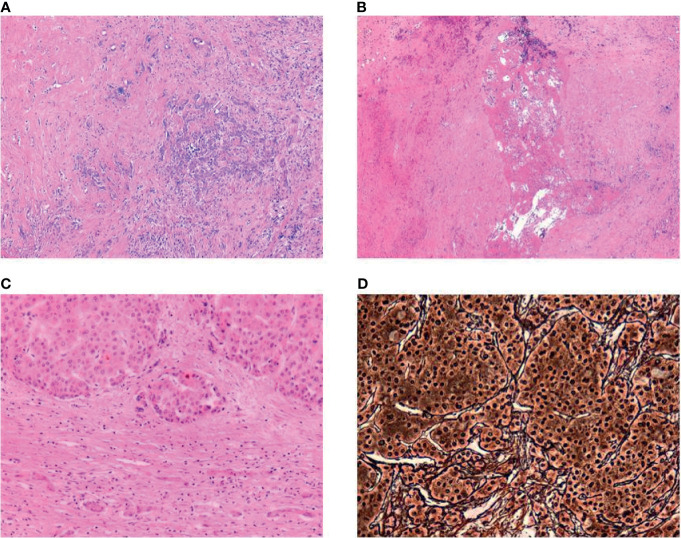
**(A)** Ductular proliferation and lymphocytic inflammation at the sclerotic border of a completely regressed HCC (H&E, magnification 50×), **(B)** Central necrosis with surrounding scarring fibrous tissue, no vital residual tumor analogous to Dworak regression score of IV (H&E, magnification 50×), **(C)** Residual HCC with dense fibrous capsule (H&E, magnification 100×), **(D)** Reticulin staining showing loss of the reticulin meshwork and macrotrabecular architecture in HCC (Gomori’s Reticulin, 200×).

Histological examination of the explanted liver was performed by an experienced pathologist. The diagnosis of an HCC was made in accordance to the criteria established in the WHO classification (13). Each respective HCC lesion was extensively sampled. The area of tumor necrosis was evaluated microscopically and expressed as a percentage of total tumor volume (**Figure 2**). Additionally, tumor regression was measured in analogy to the Dworak grading of tumor regression in rectal cancer (14).

### Statistical Analysis

Statistical analysis was performed using SPSS software (version 26.0.0.1; SPSS, Chicago, IL, USA). Descriptive statistics were performed on clinical patient data, and values were given in median and range. We determined time from enlisting to LT, and time to LT, which was defined as the time interval between the local ablative treatment (BT or SBRT) to LT.

## Results

### Patients

A total of 14 patients (male=9; female=5) were identified, of whom seven underwent HDR-BT for bridging before LT and seven were treated with SBRT. All patients had the diagnosis of liver cirrhosis, of which seven were induced ethyltoxic, three were due to a hepatitis infection, and four had other causes. The median liver volume was 1,541 ccm (range 833–3,566 ccm). Most patients had a Charlson comorbidity index (CCI) of 6 or 7 (n=11); the Child-Pugh Score (CPS) before local treatment was 5–7 points in 12 patients, and 8 and 9 points in one patient, respectively, details are listed in **Table 1**. Diagnosis of HCC was usually based on radiographic findings. However, five HDR-BT patients had a biopsy taken within the same session before undergoing treatment. Moreover, eight patients underwent tumor-specific treatments for HCC prior to SBRT (n=6) or HDR-BT (n=2).

**Table 1:** Patient characteristics.

Age	57 (31–64) years
**Gender**	
Male	9
Female	5
**Liver volume (ccm)**	1,541 (833–3,566)
**Liver cirrhosis**	
Yes	14
No	0
**Etiology**	
Ethyltoxic	7
HepB/C	3
Others	4
**Charlson comorbidity index**	
CCI 5	1
CCI 6	5
CCI 7	6
CCI 8	1
CCI 9	0
CCI 10	1
**Pretreatment Child-Pugh-Score**	
CPS 5	6
CPS 6	3
CPS 7	3
CPS 8	1
CPS 9	1
**Pretreatment AFP level (ng/ml)**	
<20	10
20–100	2
101–1,000	0
>1,000	1
NA	1
**Biopsy prior to local treatment**	
BT group	5/7
SBRT group	0/7
**Prior treatments**	
BT group	2/7
SBRT group	6/7

### BT

Seven patients were treated with HDR interstitial BT as bridging before liver transplantation. Of these, six patients received treatment of one target volume, while one patient was treated for three lesions in one treatment session. In 71% (5/7) of cases, only one BT catheter was placed, while two or four catheters were placed in the two remaining patients. The CTV had a median volume of 3.4 ccm (range 1.2–31.6 ccm). The treatment dose was prescribed to the D100 (dose covering 100% of the target volume) with 15G y, which was met in all but one patient in order to meet OAR constraints. Median liver exposure to 5 and 10 Gy was 173 ccm (range 34–606 ccm) and 11 ccm (range 12–219 ccm), respectively.

### SBRT

Seven patients received SBRT as bridging before LT. Prior to SBRT, five patients underwent CT-guided implantation of one to three fiducial markers, and four patients underwent TACE prior to SBRT. The GTV had a median volume of 4.8 ccm (range 0.8–98.1 ccm). Six out of seven patients were treated with 37 Gy (65%-iso) in three fractions, and one patient was treated with 54 Gy (100%-iso) in nine fractions. The median liver exposure to 5 and 10 Gy was 637 ccm (range 154–1,289 ccm) and 475 ccm (range 132–706 ccm), respectively.

### Toxicity

One patient experienced a CTCAE grade 3 bleeding after explantation of the catheter used for interstitial brachytherapy, and embolization had to be performed. Moreover, one patient receiving SBRT had an acute CTCAE grade 3 liver failure within 3 weeks after treatment. He subsequently underwent LT (251 days after SBRT) and was still alive at the time of our evaluation. This patient transitioned from a CPS A score at the time of treatment to a CPS C score in 82 days post-treatment. Regarding the other patients, two transitioned from a CPS A score to CPS B, while the remaining nine patients had an unchanged CPS. In one patient the blood count was not available between the local ablative treatment and LT.

### Patient Follow-Up

SBRT treatments were performed between 12/11 and 03/17 and BT treatments between 07/17 and 11/18. The median follow-up for patients receiving SBRT was 41 months (range 8–96), and the median follow-up for patients receiving BT was 17 months (range 12–26). Overall, four patients died, two in the BT group and two in the SBRT group. Of these, two patients died due to transplant rejection, one patient had a heart attack, and one patient died due to metastatic disease. No hepatic recurrences were seen; one patient developed distant metastases from HCC.

The time from enlisting to LT for the overall group was in median 228 days (range 130–4,473); in the BT group, 169 days (range 130–4,473) and in the SBRT group 242 days (range 184–1,226). Overall, the time to LT was in median 193 days (range 44–775). For patients undergoing BT, it was in median 152 days (range 47–311) and for patients undergoing SBRT 202 days (range 44–775).

Local control was assessed radiologically before LT in all cases. Of these, 10 cases were rated as treatment response (six in BT group, four in SBRT group). In one patient receiving BT and three cases of the SBRT group, the radiological report stated stable disease.

### Histological Classification

Of 14 explanted livers, 12 were available for retrospective histological examination. In two SBRT cases, unfortunately, no liver specimen was available. Overall, in eight cases, no viable tumor was found in the explanted liver. In four liver specimens, vital tumor was found as shown in **Table 2**, with Dworak regression grade 1 in two cases, and 2 or 3 in one case each. All of these were treated with BT.

**Table 2:** Dose prescription and histological classification.

	Age	Gender	Target	Prior	CTV	Catheters	Prescribed	Time to	Vital	Dworak
**BT**	(yrs)		No.	treatments	(ccm)		Dose (Gy)	Tx (days)	tumor (%)	regression score
1	62	Female	1	No	31.59	2	15 (D100)	296	80	1
2	64	Male	3	Yes	1.51; 9.78; 1.2	4	15 (D100)	311	10	3
3	57	Male	1	No	27.19	1	15 (D100)	127	70	1
4	58	Male	1	No	2.57	1	15 (D100)	152	0	4
5	47	Male	1	Yes	3.35	1	15 (D100)	47	0	4
6	59	Male	1	No	23.42	1	15 (D100)	250	0	4
7	54	Male	1	No	3.04	1	15 (D100)	115	35	2
					PTV	Combined				
**SBRT**					(ccm)	SBRT+TACE				
8	64	Male	1	No	45.4	0	9 × 6 (100%)	251	NA	NA
9	56	Female	1	Yes	109	0	3 × 12.5 (65%)	54	NA	NA
10	63	Female	1	Yes	91.3	1	3 × 12.5 (65%)	184	0	4
11	57	Female	1	Yes	220.4	1	3 × 12.5 (65%)	202	0	4
12	56	Male	2	Yes	67.5; 47	1	3 × 12.5 (65%)	44	0	4
13	31	Female	1	Yes	43.2	1	3 × 12.5 (65%)	237	0	4
14	57	Male	1	Yes	17.7	1	3 × 12.5 (65%)	775	0	4

NA, not applicable.

## Discussion

Although HCC is a radiosensitive tumor (15, 16), radiation treatment has not traditionally been considered a promising option for the treatment of HCC due to the potential injury from radiation exposure to a liver with preexisting liver cirrhosis or chronic liver disease (17). However, the introduction of SBRT has increased the precision of the radiation treatment by maximizing the dose delivered to the tumor while significantly reducing the liver exposure of non-involved healthy liver tissue. The increasing use of SBRT has led to accumulating evidence suggesting that SBRT is an effective and safe treatment option for HCC. Other, more elaborate radiation-based techniques, such as proton radiotherapy (18) or MR-guided radiation therapy (19, 20), are currently emerging in the treatment of liver tumors. However, access to these treatments is still limited today. In contrast, BT is a well-established radiation technique, which is widely available and established. We introduced a multidisciplinary workflow for interstitial BT of the liver in our institution in 07/2017. All cases that received SBRT as a bridging therapy were treated before 07/2017, which is why the follow-up is significantly longer in the SBRT group. In contrast to SBRT, which was performed non-invasively in cases of prior TACE in 29% (other patients required fiducials), BT involves a minimally invasive procedure. However, BT has been shown to be a safe treatment option, with similar results as SBRT. Moreover, BT was performed in a multidisciplinary approach and is therefore considered as a treatment option early in the decision-making process. In this regard, BT might be more competitive than SBRT compared to other LAT. To the best of our knowledge, this is the first report from a center on the clinical experience in the application of both SBRT and HDR-BT for bridging before transplant in patients with HCC. Several case series of patients treated with SBRT as bridging for transplant have been published. However, the comparison of these series is difficult because a variety of dose fractionations were used, as listed in **Table 3**. The results of an interim analysis of a prospective phase I/II study that included 60 patients, of whom 23 patients subsequently underwent LT, have been reported (21). In this study, patients with a CPS A and B were treated with a median dose of 44 and 40 Gy in three and five fractions, respectively. No relationship was reported between doses delivered to normal liver tissue, but there was an association between toxicity and CPS. Specifically, the authors reported on four patients with a Child-Pugh score of ≥8 points who experienced progressive liver dysfunction following SBRT. Therefore, the authors suggested that SBRT treatment in patients with a CPS ≥8 should be limited to patients already listed for LT. In our cohort, one patient had a liver failure grade 3 following SBRT. At the time of treatment, this patient had a CPS A score. However, the retrospective analysis of this case showed that this patient had a CPS C score at a prior visit. As LAT was denied at this point, the patient was treated to recompense liver function in order to qualify for treatment. This patient was treated with 9 × 6 Gy (100%-isodose). Another patient was treated with HDR-BT with a CPS of 9 points who underwent LT 159 days after treatment and did not experience any severe toxicity. Similarly, other authors reported on case series including patients with CPS C without reporting severe toxicities (22, 31). One retrospective review reported specifically on 12 patients with severe cirrhosis who had CPS ≥8 and were treated with SBRT as bridging to LT (30). A relatively low treatment dose with a median of 40 Gy (90%-iso) in five fractions was applied. With this treatment, however, the authors reported no case of RILD. Taken together, the treatment of patients with advanced cirrhosis appears feasible if practiced with caution. Further evidence is needed to specify dose constraints for liver exposure and dosing regimens in this subgroup of patients.

**Table 3:** Literature on radiation-based therapy as bridging to transplant.

Author	RT technique	Median dose (Gy)/fractions	Patients treated with SBRT or BT	Endpoints	Pathology	≥G3 toxicity	Liver constraints
			Total/transplanted				
Andolino et al. (21)	SBRT	40–44/3–5	60/23	LC, TTP, PFS, and OS	NA	21 × hematologic/hepatic toxicity G3	CPS A: 1/3 uninvolved liver ≤10 Gy
							V7 Gy≤500 cc
						of these 17 had preexisting G2 toxicities	CPS B: 1/3 uninvolved liver ≤18 Gy
							V12 Gy≤500 cc
Katz et al. (22)	SBRT	50 (80%-iso)/10	18/12	Histological response	2 lesions had 100% necrosis	None	V27 Gy <70%
					3 lesions had >50% necrosis		
					4 lesions had <50% necrosis		
					2 lesions had no necrosis		
O’Connor et al. (23)	SBRT	51 (50–66%-iso)/3	10/10	OS, DFS, acute toxicities, pathological response	3 lesions had no viable tumor	None	Liver-PTV V15 Gy < 35%
					3 lesions had millimetric foci of viable tumor		V15 < 700c cm
					5 lesions had residual tumor		
Mohamed et al. (24)	SBRT, yttrium-90 radio-embolization, TACE, RFA	50 (80%-iso)/5	24/14	Radiological and pathological response and DFS after LT, acute toxicity	4 lesions had 100% necrosis	None	V27 Gy < 70%
					6 lesions had >50% necrosis		
					2 lesions had <50% necrosis		
					2 lesions had no necrosis		
Guarneri et al. (25)	SBRT	40 (80%-iso)/5 or 48 (80%-iso)/3	8/8	Radiological response, pathological response, acute and late toxicities	8 lesions had CR	1× non-classic RILD	Liver volume -PTV mean dose 15 Gy (> or < 700 ccm)
					2 lesions had minimal pathological response		
					2 lesions had SD		
Moore et al. (26)	SBRT	54 (95%-iso)/3-5	23/11	OS, PFS, pathological response	3 patients had CR	1× RILD	V5 Gy <50%, V7 Gy <30%, V15 Gy <700 ccm
					6 patients had PR		for Child B mean liver dose <10 Gy
					2 patients had SD		
Sapisochin et al. (27)	SBRT, TACE, RFA	Not specified	36/30	Survival, pathological response	26 patients had some degree of tumor necrosis	None	Not specified
					4 patients had complete necrosis		
Rubinstein et al. (28)	SBRT, TACE, ETOH, RFA, MWA, SIRT	Not specified	2/2	Radiological response, pathological response	2× <80% pathological response	Not specified	Not specified
Uemura et al. (29)	SBRT	45/5	22/19	Pathological response	5 cases had CR	None	Predicted functional liver volume (pFLV)
					2 cases had PR		V16 Gy <35% (4 fractions)
					3 cases had minor necrosis		V18 Gy <35% (5–6 fractions)
					8 cases had no necrosis		
Gresswell et al. (30)	SBRT	40 (90%-iso)/5	12/11	Clinical outcome and toxicity	5 patients had CR	None	Predicted functional liver volume (pFLV)
					2 patients had extensive necrosis		V16 Gy <35% (4 fractions)
					1 patient had residual disease		V18 Gy <35% (5–6 fractions)
Denecke et al. (12)	BT, TACE	18.9/1	12/12	Matched-pair analysis	4 patients had complete/near total necrosis	None	Not specified
					7 patients had partial necrosis		
					1 patient had no necrosis		

LC, local control; TTP, time to progression; PFS, progression-free survival; OS, overall survival; CR, complete response; SD, stable disease; PR, partial response; NA, not applicable.

In our case series, one patient experienced a grade 3 bleeding needing intervention after removal of BT catheters. This is a rare complication that can occur after invasive treatment. Mohnike et al. have reported on a series of 343 interventions with a total of 1,275 catheter placements in patients with primary or secondary liver malignancies. They reported an overall major complication rate of below 5% and specifically five cases of grade 3 bleeding. All of these events occurred in patients with preexisting liver cirrhosis (32). Similarly, in a series of 781 interventions (669 of which were BT), they reported nine cases of severe bleeding (33) with an all-cause 30-day mortality rate of 1.2%. Although bleeding is overall a very rare complication, patients with liver cirrhosis are at risk and require close monitoring after interventions such as invasive BT. Notably, five of the patients undergoing HDR-BT had biopsy taken at the time of treatment with no further bleeding complications. In patients with HCC, diagnostic workup does not necessarily include biopsy due to the risk of bleeding. However, additional information acquired *via* histological assessment might be beneficial for treatment decisions for these patients in the future. Patients who are scheduled to undergo HDR-BT might therefore undergo biopsy at the same intervention without further risk of bleeding.

Regarding the fractionation schedules, **Table 3** gives an overview of the different fractionation schemes used in the literature. A retrospective analysis reported on 23 patients undergoing SBRT with risk-adapted doses of 30, 48, and 54 Gy (95%-iso) in five, four, and three fractions (26). In our cohort, all patients undergoing SBRT were treated with 37 Gy (65%-iso) in three fractions except for one patient with poor liver function, who was treated with 54 Gy (100%-iso) in nine fractions. All patients undergoing BT were treated with 15 Gy in a single fraction. There is a need to homogenize dose regimens for patients undergoing SBRT treatments of the liver in order to achieve comparable results. To date, our cohort is not large enough to support the concept of risk-adapted dose prescription according to the CPS.

In our cohort, the local control was assessed radiologically in all cases before LT. Overall, 10 patients were radiologically rated as locally controlled, and in four patients, hepatic progress could not be ruled out, however, without clear signs of a progression. Therefore, all cases were classified as at least stable disease and were reviewed in a dedicated LT board. All cases treated with SBRT in our cohort had no viable tumor in explanted liver, while some degree of residual tumor was found in four of seven patients treated with BT, which might be influenced by the shorter intervention-to-LT interval in BT. **Table 3** gives an overview on the available studies and pathological results using SBRT or BT as bridging for LT. Overall, no correlation could be found with time to transplantation.

To date, no randomized controlled trials have been conducted to compare SBRT or BT to other LAT, particularly in the context of bridging before LT. Comparative data are scarce, with only few centers reporting their experiences. One study including 60 patients undergoing SBRT, TACE, RFA, or yttrium-90 as bridge to LT reported on comparable radiological response of all four treatment modalities with superior acute toxicity of SBRT and yttrium-90 over TACE and RFA (24). However, in this study, patients with elevated pretreatment bilirubin level and CPS C were allocated to receive SBRT, which implies a negative selection for these patients. Patients with small tumors (<3 cm) received RFA, and larger-volume HCCs were treated with yttrium-90. In another large intention-to-treat comparison, 406 patients who underwent either SBRT, TACE, or RFA as bridging before transplantation were compared (27). In this cohort, patients received SBRT if they were ineligible for TACE or RFA due to poor liver function, thrombocytopenia, or because of technical limitations. Overall, 36 patients were treated with SBRT, all of which subsequently had LT. The authors concluded that SBRT was as effective and safe as TACE or RFA. BT as bridging before LT has been described by one other group so far (12). In this publication, 12 patients who underwent BT before LT were evaluated in a matched-pair comparison with patients treated with TACE. The results of this retrospective single-center study indicates that with BT, comparable results in terms of pathological response and disease-free survival can be achieved as in patients treated with TACE. The authors pointed out that TACE as well as RFA had limitations concerning the size of treated lesions. Local control is inversely correlated to tumor size. Therefore, the authors highlight that BT might be able to fill the gaps of indications. Notably, in this study BT was performed with a dose of 15–25 Gy (mean dose 18.9 Gy), which is higher than the doses we applied.

Regarding the limitations of the current study, all evaluations were performed retrospectively with a relatively small number of patients. However, since the published data on HDR-BT as bridging to liver transplant are very limited, we believe it is important to report our experience. Although we perform HDR-BT in about 300 liver lesions per year, there is still a negative selection of patients, especially in those listed for LT. Only patients not perfectly suitable for other local treatments receive either HDR-BT or SBRT; therefore, the results in this report might also represent a negative preselection. In the future, SBRT performed with hybrid MR-Linac systems will further enrich the treatment options for these patients. With the possibility of automated respiratory gating provided by the use of online MR and the superior imaging quality, the PTV margins can be reduced to a minimum. First reports are promising (20), and research in this field is ongoing.

## Conclusion

The use of radiation-based treatments such as SBRT and BT as bridging to transplant is feasible, with few cases of RILD reported in the literature. Our own experience with both SBRT and BT contributes to the growing body of knowledge and proofs that radiation-based treatments can close a gap for patients not fit for other treatment options. However, further evidence is needed to homogenize radiation dose exposure to normal liver tissue and provide guidance for treatment modifications in patients with more advanced liver cirrhosis.

## Data Availability Statement

The original contributions presented in the study are included in the article/supplementary material. Further inquiries can be directed to the corresponding author.

## Ethics Statement

The studies involving human participants were reviewed and approved by the Ethics Committee, LMU Munich. Written informed consent for participation was not required for this study in accordance with the national legislation and the institutional requirements.

## Author Contributions

FW participated in patient treatment, data acquisition, data analysis, and in drafting the manuscript. FF, SG, and MR participated in data acquisition and data analysis. RE and JN performed the histological classification. MG, ET, MS, JR, and CB participated in treating the patients and critically reviewed the manuscript. SC participated in data acquisition, data analysis, and drafting the manuscript. LN participated in patient treatment, data acquisition and data analysis. All authors contributed to the article and approved the submitted version.

## Conflict of Interest

SC has received a research grant from Elekta.

The remaining authors declare that the research was conducted in the absence of any commercial or financial relationships that could be construed as a potential conflict of interest.

## Publisher’s Note

All claims expressed in this article are solely those of the authors and do not necessarily represent those of their affiliated organizations, or those of the publisher, the editors and the reviewers. Any product that may be evaluated in this article, or claim that may be made by its manufacturer, is not guaranteed or endorsed by the publisher.
